# Sound production in female *Trichopsis schalleri* (Labyrinth fishes): comparison to males and evolutionary considerations

**DOI:** 10.1080/09524622.2018.1555773

**Published:** 2018-12-18

**Authors:** Friedrich Ladich, Günter Schleinzer

**Affiliations:** Department of Behavioural Biology, University of Vienna, Vienna, Austria

**Keywords:** Acoustic signals, sound level, dominant frequency, sex-specific differences, sexual dimorphism, sonic organs

## Abstract

Croaking gouramis (genus *Trichopsis*, Anabantoidei) generate series of two-pulsed bursts (croaks) during agonistic interactions. Sex-specific differences are minor in *T. vittata* which raises the question whether sexes differ in the other two species. The current study analyses sounds recorded in female *T. schalleri*, compares the sound characteristics to those of males investigated earlier and correlates these characteristics to female body size. Sex-specific differences were found in three out of six sound characteristics. In females, sounds were lower in burst number, burst period and SPL. Pulse period, dominant frequency and peak-to-peak amplitude ratios of pulses did not differ between sexes. Burst period and SPL increased significantly with female body weight, whereas dominant frequency decreased. The present acoustic data indicate the sex-specific differences are more pronounced in *T. schalleri* than *T. vittata*. The results also demonstrate that both sexes are vocal, which remains to be shown for females of the third species, *T. pumila*, which have poorly developed sonic organs. The evolution of the pectoral sound-producing mechanism in *Trichopsis* is most likely based on an exaptation process during which acoustic signals are generated by fin tendons initially related to other functions as is evident in closely related genera lacking this organ.

## Introduction

Acoustic signalling during social behaviour has been described in representatives of several dozen families of bony fishes. Most studies showed sound production in males, occasionally in females and in several cases the sex of the caller was unknown (for reviews see Myrberg 1981; Amorim 2006; Ladich and Myrberg 2006; Myrberg and Lugli 2006, Ladich 2015a). Sounds have rarely been recorded under identical conditions in both sexes, making comparisons of sound properties difficult (Lagardere et al. 2005; Ladich 2007; Ueng et al. 2007; Simoes et al. 2008; Tellechea et al. 2010; Oliveira et al. 2014; Fine and Waybright 2015). The lack of sound recordings and analyses in females can be explained by the fact that the males typically occupy territories and defend nest sites when seeing an intruder by emitting vocal, territorial keep-out signals (Myrberg 1997; Amorim et al. 2015); females were seldom investigated in this context.

Sound-generating mechanisms are generally present in both sexes of vocal species except for a few sciaenids (drums or croakers) such as the spot *Leiostomus xanthurus* and the weakfish *Cynoscion regalis* (Hill et al. 1987). This indicates that females are acoustically active in almost all vocal species. Sonic organs in fishes are always sexually dimorphic and typically smaller in females (Templeman and Hodder 1958; Courtenay 1971; Kratochvil 1978; Fine et al. 1990; Bass 1992; Connaughton and Taylor 1995; Kéver et al. 2012; Casaretto et al. 2016). Behavioural observations in numerous species show that females often defend territories vigorously and produce aggressive sounds similar to males (Myrberg et al. 1965; Ladich 1990, 2007; Simoes et al. 2008; Hadjiaghai and Ladich 2015).

In which behavioural context do females and males produce sounds so that the sexes can be compared unequivocally? Most often both sexes produce sounds during agonistic interactions or in distress situations, eg when defending their territories or when hand-held, allowing intersexual comparison of sounds emitted under similar circumstances. Rarely do both sexes vocalize during courtship and spawning. Male and female seahorses (genus *Hippocampus*, Syngnathidae) reportedly produce clicking sounds during courtship (Anderson 2009; Oliveira et al. 2014). More often only male fishes vocalize during reproduction, eg to advertise their nest sites or to attract females (for reviews see Lugli et al. 1997; Myrberg and Lugli 2006; Amorim et al. 2015), with one exception in which only females vocalize (Ladich 2007).

The anabantiform (labyrinth fish) genus *Trichopsis* (croaking gouramis) evolved a unique pectoral sound-generating mechanism which is unknown in closely related genera (Kratochvil 1985). The mechanism consists of enlarged pectoral fin muscles which stretch two thickened pectoral fin tendons. Plucking these tendons during rapid pectoral fin beating produces series of double-pulsed bursts known as croaking sounds, which are regularly audible during agonistic interactions (Kratochvil 1978). Interspecific differences in the anatomy of this sonic organ were described between the croaking gourami *T. vittata* and the pygmy gourami *T. pumila* (Kratochvil 1978, 1980), whereas data on the third species, the threestripe gourami *T. schalleri*, are lacking. The acoustic signals produced by males of all three species during agonistic interactions clearly differ in their temporal, spectral and intensity characteristics (Ladich et al. 1992). Interestingly, the differences in these sounds are paralleled by differences in agonistic behavioural patterns (Bischof 1996). This indicates that the anatomical and acoustical signal differences may be linked to those in fighting behaviour.

Earlier anatomical investigations revealed furthermore that sound-producing structures are sexually dimorphic, being larger in males in *T. vittata* and *T. pumila*, but the intersexual difference in sonic organs is much smaller in *T. vittata* than in *T. pumila* (Kratochvil 1980). A subsequent study in *T. vittata* showed that male and female agonistic sound features resemble each other (Ladich 2007), which reflects the anatomy of this species. In contrast, the small size of female sonic organs in *T. pumila* raises the question if females are able to signal acoustically (Marshall 1966; Kratochvil 1980).

The relationship between sound characteristics and body size has very rarely been investigated in female fish (black drum *Pogonias chromis* – Tellechea et al. 2010; piranhas – Mélotte et al. 2016; *T. vittata* – Ladich and Maiditsch 2018). Temporal, spectral and intensity characteristics may be correlated to body size, size of sonic organs or age, as has frequently been demonstrated in males as well as juveniles in ontogenetic studies. The correlation most often found is a negative one between fish size and the dominant frequency of pulsed sounds in numerous unrelated taxa (eg male bicolor damselfish *Stegastes partitus* – Myrberg et al. 1993; both sexes of the drum *P. chromis* – Tellechea et al. 2010; male *T. vittata, schalleri* and *pumila* and juveniles *T. vittata* – ; Ladich et al. 1992; Henglmüller and Ladich 1999; juvenile red gurnards *Eutrigla gurnardus* – Amorim and Hawkins 2005; juvenile squeaker catfish *Synodontis schoutedeni* – Lechner et al. 2010; juvenile Lusitanian toadfish *Halobatrachus didactylus* – Vasconcelos and Ladich 2008; reviewed in Ladich 2015b). The sound pressure level (SPL) was rarely reported to depend on size or age in adults and juveniles (juveniles reviewed in Ladich 2015b; adult croaker *Cynoscion regalis* – Connaughton et al. 2000; callichthyid catfish *Hoplosternum thoracatum* – Hadjiaghai and Ladich 2015). The temporal patterns of sounds such as duration or pulse periods generally increase with size or age in juveniles and adults (eg skunk clownfish *Amphiprion akallopisos –* Colleye et al. 2009; catfish *H. thoracatum* – Hadjiaghai and Ladich 2015), but opposite trends were also reported.

The present investigation pursues three aims: (1) to analyse sounds produced by female *T. schalleri* during agonistic interactions and compare them to male sounds published by Ladich et al. (1992), (2) to correlate sound characteristics and body weight in females and (3) draw conclusions on the anatomy, sexual dimorphism and the evolution of sonic organs in anabantoids.

## Methods

### Animals

Twenty female *T. schalleri* (0.47–1.19 g) purchased from a local pet supplier were investigated. They were kept in 50–300 l community tanks before introducing them into a test tank. Aquaria were planted but not aerated or filtered because labyrinth fishes possess air-breathing organs (suprabranchial organs or labyrinths) dorsally of the gills (Richter 1988). The temperature was kept at 28 ± 1°C and a 12 h:12 h light:dark cycle was maintained. Fish were fed *Tubifex* worms, chironomid larvae or *Daphnia* spp.

### Experimental setup and sound recordings

The test tank was 60 × 35 × 40 cm and equipped with plants, a half flower pot as a hiding place and a plastic sheet separating the tank in two halves. The temperature and light rhythm were similar to the holding tanks. One female was introduced into each half of the test tank for 2 days to adapt and become territorial. After removing the separating sheet, the fish defended their territory and vocalized.

Acoustic signals were recorded using a hydrophone (Brüel & Kjaer 8101, sensitivity

−184 dB re 1 V/μPa) placed in the centre of the tank close to the back wall. The hydrophone was connected to a microphone power supply (Brüel & Kjaer 2804) and to a tape recorder (UHER Report Monitor). Data from 20 male *T. schalleri* taken from the study by Ladich et al. (1992) were used for the comparison between sexes. Males and females were recorded at approximately the same time.

### Sound analysis

The sounds recorded were analysed using a Gould 1602 storage oscilloscope and S-Tools, the Integrated Workstation for Acoustics, Speech and Signal Processing developed by the Acoustics Research Institute of the Austrian Academy of Sciences in Vienna. All sounds were digitized using a sampling rate of 16 kHz.

**Figure 1.**
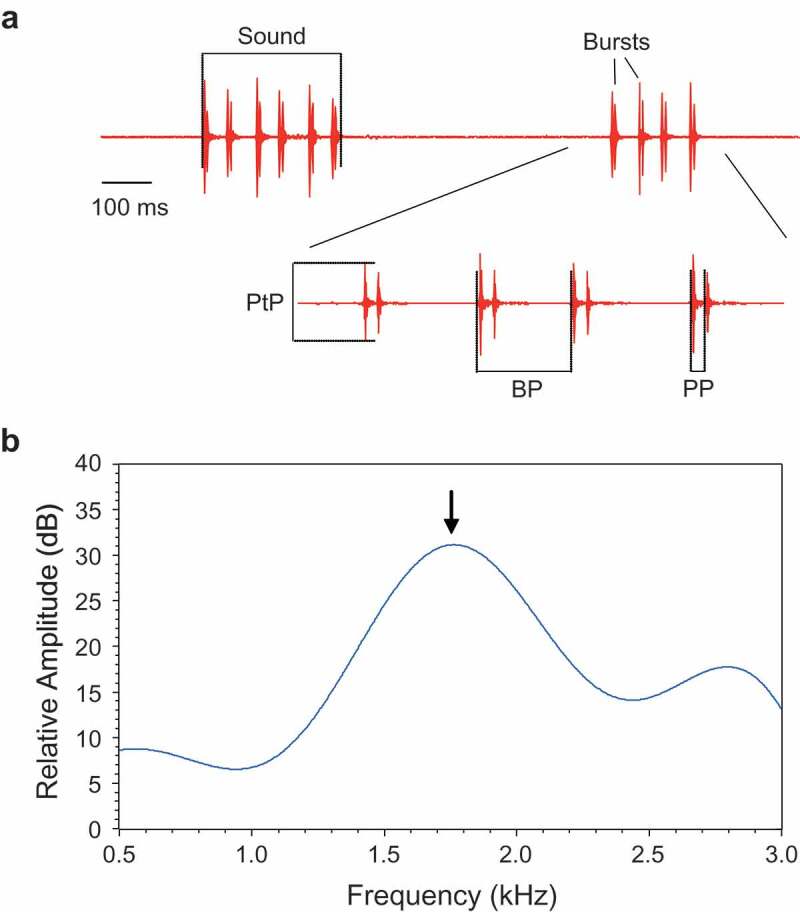
(a) Oscillograms of two female croaking sounds consisting of 6 and 4 bursts, and an expansion of the second sound illustrating the sound characteristics analysed. BP – burst period, PP – pulse period, PtP – Peak-to-peak amplitude of first pulse within a burst. (b) Cepstrum-smoothed power spectrum of a female sound. Arrow indicates the dominant frequency. Sampling rate 16 kHz, filter bandwidth 5 Hz, number of coefficients 15, 75% overlap, Hanning window.

The following sound characteristics were determined for each female (see also methods in Ladich et al. 1992; Ladich 2007). (1) Number of bursts within a sound. (2) Burst period, defined as the time interval between the onsets of two consecutive bursts (Figure 1(a)). (3) Pulse period, defined as the time between the onsets of two successive pulses within a double-pulsed burst. (4) The dominant frequency of sounds, determined by calculating the cepstrum smoothed power spectrum using S-Tools (Figure 1(b), the (5) Peak-to-peak amplitude ratio of two consecutive pulses within a double-pulse burst (Figure 1(a)).

### Sound pressure level determination

**Figure 2.**
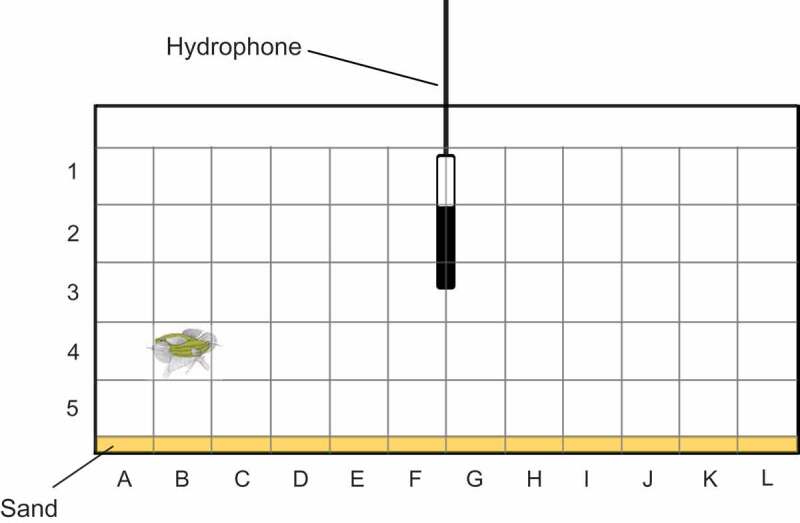
Experimental tank showing the grid used for SPL measurements and calculations. Sectors are labelled by numbers and letters. For clarity, the third position depth (front, middle and back of tank) is not included in this graph. Two gouramis are shown during an agonistic interaction in the sector B4front.

Because of the varying distance between fish and the hydrophone, the test tank was divided into 180 sectors and the position of each vocalizing female noted (length: 12 positions; height: 5 positions; depth: 3 positions – front, middle and back) (Figure 2). The SPL of the female sound was measured in dB re 1 µPa using a measuring amplifier (Brüel & Kjaer 2606, scale SA 0197, RMS Fast time weighting, linear frequency weighting between 22.5 Hz and 22.5 kHz). To correct for different distances between the hydrophone and calling females, a correction factor was calculated. One typical fish sound was played back at constant SPL (120 dB at 25 cm distance from the hydrophone) from a small loudspeaker (Fuji 7G06, 8 Ohm, 0.8 W) in each of the 180 sectors and the SPL noted. The difference between the SPL values measured while a female produced sounds in a particular sector and the SPL value of the speaker in this sector was calculated. This difference was subtracted from the standard SPL of 120 dB when the fish’s SPL was lower than the speaker’s SPL (and vice versa). This yielded an absolute SPL value for each croaking sound which was independent of the distance from the hydrophone, of the position and of structures in the tank.

### Statistical analysis

Up to 20 sounds were analysed for each female, and the means and standard errors of sound characteristics were calculated. The means of each sound property of 20 females (and 20 males) were subsequently used to calculate the differences between sexes. Acoustic variables of females and males were tested for normality using a Shapiro-Wilk test. If data were normally distributed, an independent sample *t*-test (in all other cases Mann-Whitney U-Tests) was chosen to calculate the differences between sexes.

As all female variables were normally distributed (except pulse period), Pearson’s correlation coefficients were calculated to investigate the degree of correlations between female body weight and sound characteristics (Spearman correlation coefficient for pulse period). All calculations were done using SPSS 23 (IBM SPSS Statistics).

### Ethical note

Agonistic interactions between Threestripe gouramis consist of lateral displaying during which both sexes raise their unpaired fins, circle head to tail and produce croaking sounds, without any physical contact between opponents. As the intention of this study was to record sounds, agonistic interactions were stopped as soon as enough sounds were recorded. Any unwanted aggressive behaviour was terminated by reinserting the plastic sheet between individuals.

## Results

**Figure 3.**
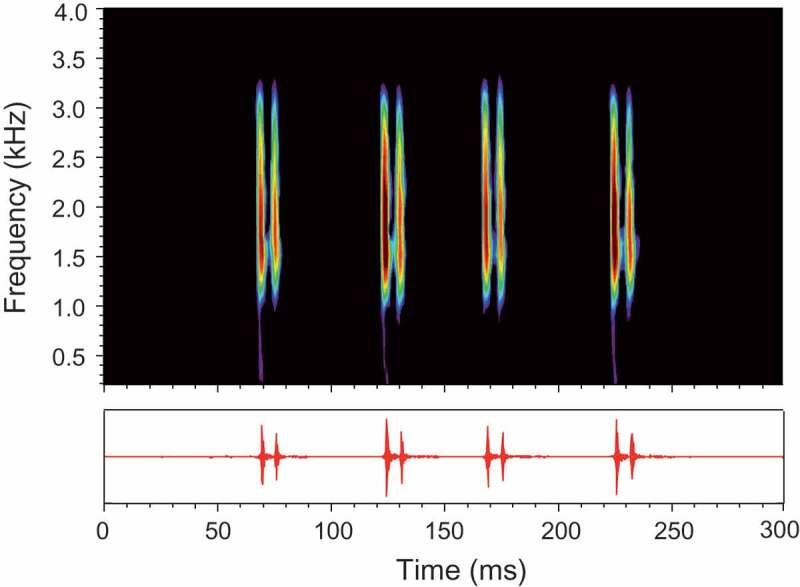
Sonogram (above) and oscillogram (below) of an agonistic sound emitted by a female *T. schalleri*. The sound consists of four double-pulsed bursts. Sampling frequency 16 kHz, filter bandwidth 180 Hz, 75% overlap, Hanning window.

Croaking sounds produced by female *T. schalleri* during agonistic interactions consisted of series of bursts typically built up of two pulses (double-pulsed burst or long burst) and occasionally of one pulse (single-pulsed burst or short burst) (Figures 1(a) and 3). The main energy was concentrated between 1.3 and 1.9 kHz.

### Sound characteristics and differences between sexes

**Figure 4.**
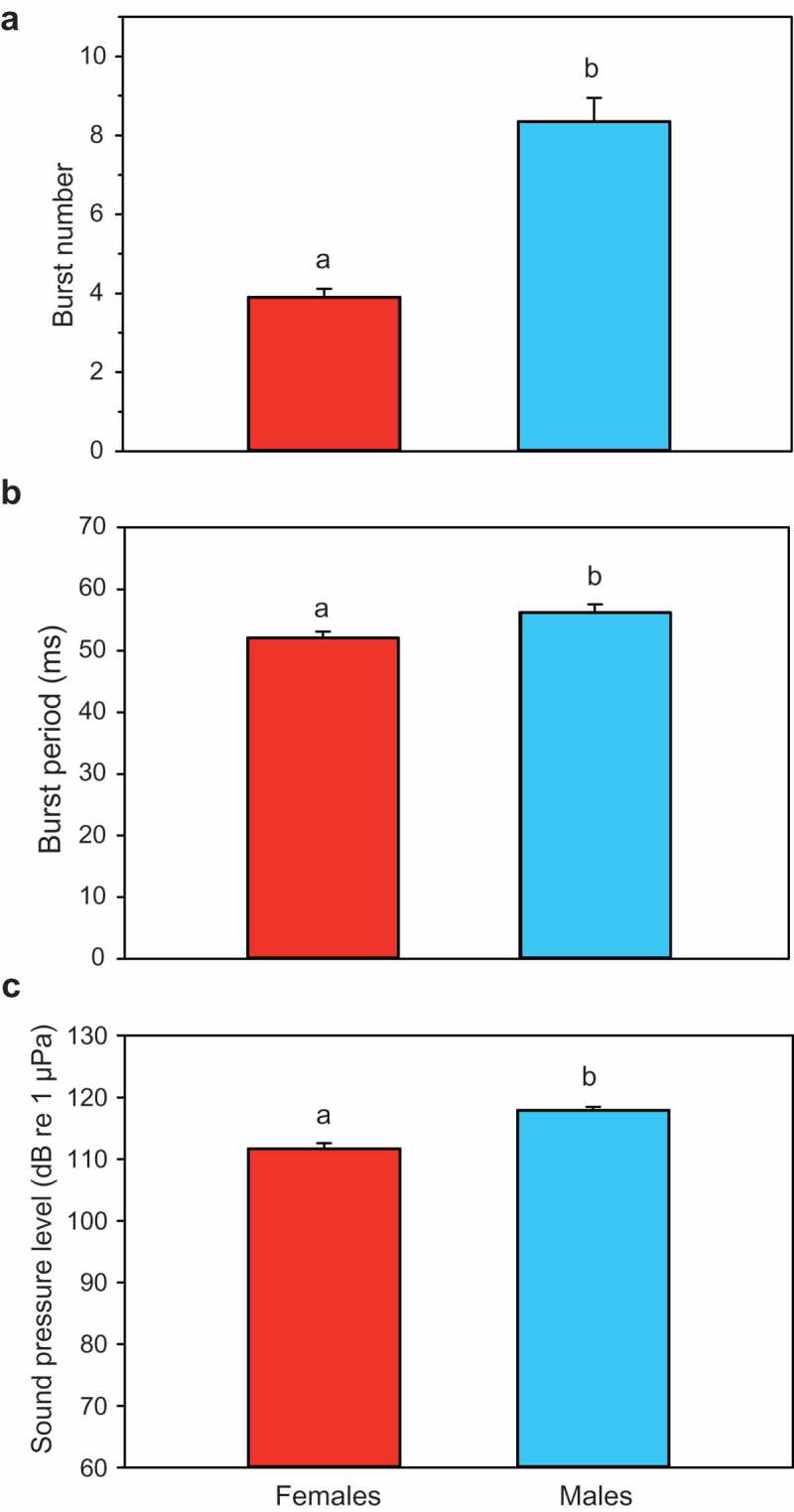
Mean (± S.E.) characteristics of agonistic sounds in female and male *T. schalleri*. (a) Burst number, (b) Burst period and (c) Sound pressure level. Different letters indicate significant differences between sexes.

Body weight did not differ between sexes (U-test, U = 132.0, n = 40, p = 0.68), but half of the sound characteristics analysed did. Two out of three temporal characteristics analysed differed between sexes. The number of bursts was significantly smaller in females than males (*t*-test, t = 7.075, df = 38, *p* < 0.001) (Figure 4(a)). Females produced on average half as many bursts per sound as males. The burst period was again also smaller in females (*t*-test, t = 2.578, df = 36, *p* < 0.02) (Figure 4(b)). Therefore, sound duration – defined as burst period times the number of bursts – was approximately half as long in females than males. In contrast, the pulse period was similar in both sexes (U-test, U = 155.5, n = 37, n. s.)

The dominant frequency did not differ between sexes (U-test, U = 141.5, n = 38, n. s.), nor did the peak-to-peak amplitude ratio between the first and second pulse within a burst (*t*-test, t = 0.525, df = 34, n.s.). The SPL was approximately 6 dB lower in females (*t*-test, t = 6.272, df = 38, *p* < 0.001) (Figure 4(c)).

### Sound characteristics and body size

**Figure 5.**
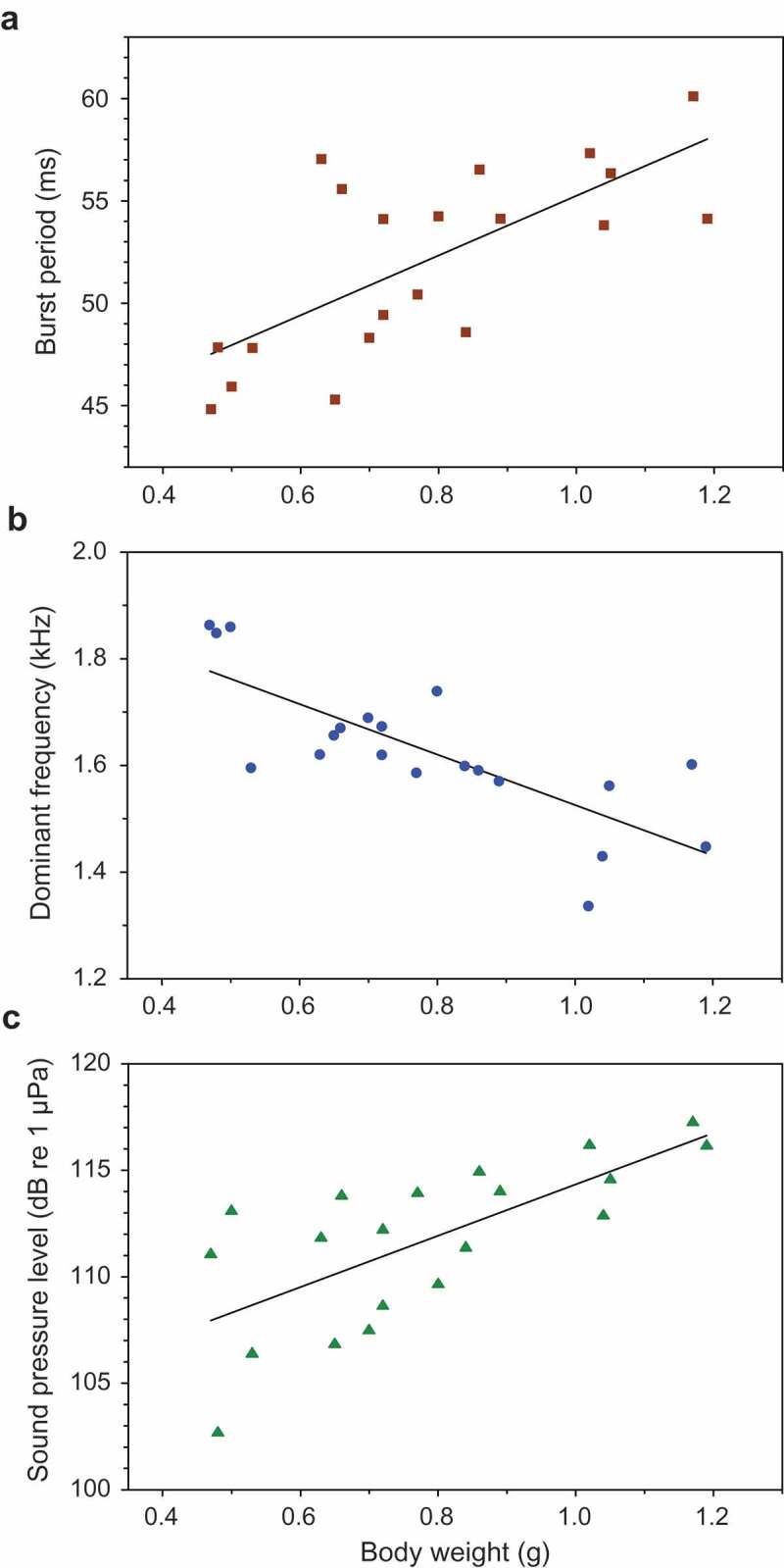
Relationship between body weight (BW) and sound characteristics in female *T. schalleri*. Regression equations (a) Burst period = 40.7 + BW *14.6. (b) Dominant frequency = 1.999 – BW*0.473. (c) Sound pressure level = 102 + BW*12.1.

Body weight was significantly correlated to three temporal, spectral and amplitude properties of female sounds. The burst period increased with body weight (r = 0.707, n = 20, *p* < 0.001) (Figure 5(a)), and the dominant frequency decreased significantly (r = −0.774, n = 20, *p* < 0.001) (Figure 5(b)). No correlation was found between weight and the peak-to-peak amplitude ratio of pulses within bursts, although it was close to significance (r = −0.439, n = 20, *p* = 0.053).

The SPL increased significantly with weight in female Threestripe gouramis (r = 0.706, n = 20, *p* < 0.001) (Figure 5(c)). SPL was furthermore positively correlated to burst number, indicating that females produce longer sounds at higher SPLs (r = 0.594, n = 20, *p* < 0.001). No correlation was found between size and pulse period or peak-to-peak amplitude ratio.

## Discussion

The few descriptions of female sounds in general and of statistically significant differences between sexes do not accurately reflect the vocal activity of female fish (Ladich 2007; Fine and Waybright 2015; Ladich and Maiditsch 2018). We can assume that in all fish species having sound-generating mechanisms, both sexes are vocal during agonistic interactions such as during territorial defence and when being disturbed by con- or heterospecifics (Ladich 2015a; Casaretto et al. 2016). More detailed anatomical analyses in toadfish, drums, cuskeels and croaking gouramis show that female sonic organs are smaller (to different degrees) than males. This affects the SPL of sounds. Sex-specific differences in the temporal patterns of sound properties are less clear. Female sounds may have longer pulses, longer pulse intervals and more pulses per call than male sounds, or the opposite may be the case, or they may not differ between sexes (Lagardere et al. 2005; Ueng et al. 2007; Simoes et al. 2008; Hadjiaghai and Ladich 2015). In summary, females have an active but insufficiently explored vocal life (Fine and Waybright 2015).

### Technical aspects

The minimum resonant frequency of the tank used for sound recording was 3.37 kHz according to the formula given by Akamatsu et al. (2002). This frequency is considerably higher than the dominant frequencies of the croaking sounds of *T. schalleri*, which were below 1.9 kHz. Thus, the original spectrum of the fish sound is not distorted by the aquarium and allows accurately characterizing the original sounds. Moreover, the dominant frequencies of sounds correspond to the best hearing bandwidth in the genus *Trichopsis*, while anabantoids in general are quite insensitive at frequencies above 3 kHz (Ladich and Yan 1998).

Sound pressure levels measured in small tanks cannot be compared directly to those potentially measurable in the field. Gouramis live in shallow standing waters in Southeast Asia with abundant vegetation such as rice fields (Richter 1988). Observations of fighting fish and measurements would be quite challenging in these habitats. Importantly, the absolute SPLs in the current study are used only for comparison between sexes and correlations to body size.

### Acoustic signalling in the genus *Trichopsis*

The genus *Trichopsis* is an ideal candidate to study the inter-specific and sex-specific evolution of signalling systems. This genus consists of only three species which have in common a unique sound-generating mechanism unknown in all other genera of the family Osphronemidae and the suborder Anabantoidei (labyrinth fishes) (Kratochvil 1985). The small number of species enables a deep insight into a taxon in terms of the evolution of acoustic signalling. Anatomical, behavioural and acoustical investigations have revealed interesting differences in the acoustic and visual signalling between males of the three species and, additionally, species-specific differences between sexes. The pectoral sonic mechanism seems to play a key role in this differentiation.

The basic difference is the variability in the sonic organs – modified pectoral fin muscles and tendons – which subsequently affect vocalizations within this genus. Males of the largest species *T. vittata* possess relatively smaller sonic muscles (Musculus adducter superficialis and profundus) (Kratochvil 1978, 1980) than males of the smallest species *T. pumila* (note that data on *T. schalleri* are missing). Surprisingly, however, the smallest species, the pygmy gourami, produces the loudest sounds. Agonistic sounds of *T. pumila* are on average 8–9 dB louder than in males of the other two species, which themselves have similar values (Ladich et al. 1992). Secondly, *T. pumila* generates high-level sounds even though the second enhanced tendon is less developed than in *T. vittata*, where both tendons are similarly enhanced (Kratochvil 1978). The anatomy of the sonic organ in the third species *T. schalleri* can only be deduced from the sounds they produce. The peak-to-peak amplitude ratio of the first and second pulse within a burst is similar to *T. pumila* and larger than in *T. vittata* (Ladich et al. 1992). This indicates that the sonic organs of male and female *T. schalleri* are similar to those of male *T. pumila* (which means that the second tendon is smaller than in *T. vittata*). Besides the potential anatomical similarity, *T. schalleri* males differ from their congenerics by emitting the longest sounds (highest number of bursts and largest burst periods) in agonistic encounters (Ladich et al. 1992).

There is another potential explanation for the rather low level and short sounds in male *T. vittata*. The largest member differs from both other species in visual signalling. While agonistic behaviour consists of a lateral display phase (LD-phase) in all three species and both sexes – during which fish spread their unpaired fins, circle in a head to tail position and produce sounds (see Figure 1(a) in Ladich 2007) – fighting proceeds to the frontal display (FD) phase in both sexes only in *T. vittata* if agonistic interactions are not decided during the LD-phase (Ladich 1998). In the FD-phase, the fish protrude their mouths towards each other and pivot 45° around a longitudinal axis (see Figure. 9(d) in Henglmüller and Ladich 1999) and do not produce any sounds. Such an FD-phase has not been observed in any sex of the two smaller species during agonistic interactions (Bischof 1996). Clearly, these three *Trichopsis* species differ in the way they signal visually and acoustically to gather sufficient information for assessing the fighting ability of opponents and resolving conflicts (Enquist and Jakobson 1986).

Beyond the clear differences in the behavioural repertoire of males during agonistic interactions and signals, the species-specific differences become even more complex when examining the sex-specific differences in acoustic signalling. In the largest species *T. vittata*, the sex-specific differences are small (Ladich 2007). Agonistic sounds have a lower SPL in females, most likely reflecting their somewhat smaller sonic organ (Kratochvil 1978; Ladich 2007). Temporal and spectral sound properties do not differ between sexes. Sex-specific differences in anatomy are more pronounced in the other two species. Sonic organs in female *T. pumila* are lacking or are much smaller than in males, resulting in contradictory conclusions. Marshall (1966) claimed that both sexes of *T. pumila* emit croaking sounds, without giving any experimental data. In contrast, Kratochvil (1980) concluded, based on his anatomical data, that females are unable to produce sounds. All female *T. schalleri* vocalize during territorial disputes, similar to female *T. vittata*, but the sounds produced differ from male sounds to a higher degree than in *T. vittata*. Female *T. schalleri* emit sounds with lower SPLs (similar to female *T. vittata*), but they are additionally shorter (half as long) than male sounds. This indicates that female *T. schalleri* have well-developed though sexually dimorphic pectoral sonic organs, enabling them to vocalize during all agonistic interactions. This situation remains unclear in *T. pumila*.

Finally, the assumption is that the species- and sex-specific variability in the development of sonic organs affects courtship behaviour. According to our current knowledge, *T. vittata* is the only fish species in which only the females vocalize during courtship and before spawning (Marshall 1966; Ladich 2007). Female pre-spawning sounds have a lower SPL and fewer bursts than female agonistic sounds (Ladich 2007). Due to the poorly (or not) developed sonic organs in female *T. pumila*, it is unlikely that they vocalize during reproduction. We hypothesize that *T. schalleri* females are vocal during courtship, but this remains to be proven.

### Correlations between size and sound properties

The most common correlation between size and sound characteristics in animals in general and fish in particular is the negative relationship between size and sound frequencies (frogs: Davies and Halliday 1978; fish: Myrberg et al. 1993; Ladich 2015b). This relationship may be important in assessing potential mates or the fighting ability of opponents (Myrberg et al. 1986; Ladich 1998). A negative correlation is given in males of all species of the genus *Trichopsis* and in females of *T. vittata* and *T. schalleri* (Ladich et al. 1992; Ladich and Maiditsch 2018; present study). Such a relationship has further been shown in females of the drum *Pogonias cromis* (Tellechea et al. 2010), potentially the skunk clownfish *Amphiprion akallopisos* (Colleye et al. 2009) and can be expected in many more species. It is lacking in the oyster toadfish *Opsanus tau* and the Padanian goby *Padogobius bonelli*, most likely because they do not produce pulsatile sounds (Fine and Waybright 2015), but also in female longsnout seahorses, which emit pulsatile sounds (Oliveira et al. 2014). The lack of a correlation may be due to the small size ranges investigated. In labyrinth fishes, the suprabranchial or labyrinth organ (SBO), an air-breathing cavity dorsal to the gills (Bader 1937), may be responsible for the dominant frequency of their broad band pulses because the SBO is located adjacent to the pectoral sound-generating organ.

The fundamental frequency of drumming sounds is controlled by the muscle contraction rate rather than the resonant frequency of air-filled cavities, and thus should not show such a size-frequency relationship according to Myrberg et al. (1993). Nevertheless, the fundamental frequency can be related to size in fish possessing drumming muscles (sciaenids: Tellechea et al. 2010; doradid catfish: Knight and Ladich 2014; four out of six piranha species: Mélotte et al. 2016). Larger muscles with longer fibres may need longer to complete a contraction, resulting in drumming muscles producing lower contraction rates in larger fish (Connaughton et al. 2000).

Correlations between size and SPL have rarely been shown in adult fish, but have been demonstrated in several ontogenetic studies in non-related taxa such as in *T. vittata*, the Lusitanian toadfish *Halobatrachus didactylus* and the African squeaker catfish *Synodontis schoutedeni* (Schneider 1961; Henglmüller and Ladich 1999; Lechner et al. 2010). A size-dependent increase in SPL was shown in male *Cynoscion regalis* (Connaughton et al. 2000) and in both sexes of *Opsanus tau* up to a weight of 200 g (Fine and Waybright 2015). In catfish species, a size-SPL relationship was observed when both sexes and several species were pooled (Knight and Ladich 2014; Hadjiaghai and Ladich 2015). No such a relationship was evident in both sexes of the longsnout seahorse *Hippocampus reidi* or in both sexes of adult *T. vittata* (Ladich et al. 1992; Oliveira et al. 2014; Ladich and Maiditsch 2018). Interestingly, a strong positive correlation is reported in female *T. schalleri* in the present study, but not in the prior study on males (Ladich et al. 1992).

Temporal characteristics of acoustic signals such as duration, number of bursts or pulses, and burst or pulse periods within sounds typically increase with size in all species studied (eg Amorim and Hawkins 2005; Connaughton et al. 2000; Colleye et al. 2009; Lechner et al. 2010; Tellechea et al. 2010; Knight and Ladich 2014; Hadjiaghai and Ladich 2015; 2 out of 8 piranha species: Mélotte et al. 2016). Exceptions include the toadfish *H. didactylus*, in which the number of pulses and thus sound duration decreased with growth (Vasconcelos and Ladich 2008). Furthermore, the sound duration may depend on the size of sonic organs and less so on total body size. Ladich (1997) and Pruzsinszky and Ladich (1998) showed that sound duration depended on the length of the pectoral spines in several catfish species. In the genus *Trichopsis* no correlations were found between temporal characteristics and size in both sexes of *T. vittata* and male *T. schalleri* (Ladich et al. 1992; Ladich and Maiditsch 2018). This contrasted to female *T. schalleri*, in which burst period and body weight showed a strong positive correlation. Interestingly, such a correlation was described for male *T. pumila* (Ladich et al. 1992).

The burst period, namely the time between the onsets of sounds produced by different pectoral fins, was positively correlated to size in female *T. schalleri*. This indicates that larger muscles may take longer to complete a twitch and, therefore, the time between subsequent contractions of pectoral fin muscles will increase with size (Connaughton et al. 2002; Fine and Parmentier 2015). The burst number is furthermore positively correlated to the SPL in females. This correlation resembles the positive relationship between number of bursts in agonistic sounds and SPL in female *T. vittata* (Ladich and Maiditsch 2018). One potential explanation is that the maximum tension of enhanced pectoral fin tendons may only be reached in longer sounds, which are subsequently louder.

### Evolution of sound production in labyrinth fishes *(anabantoids)*

What do we know about the evolution of sound production in labyrinth fishes? All members possess an air breathing organ (labyrinth organ) close to the inner ears, which in parallel enhance their hearing sensitivities (Schneider 1942; Yan 1998). This SBO potentially facilitates acoustic communication and may have been involved in the occasional production of (most likely) pharyngeal teeth sound in the genera *Colisa, Macropodus* and *Belontia* (Kratochvil 1985, Schuster 1986). Nonetheless, a well-developed sonic organ is known only in the genus *Trichopsis. Trichopsis* belongs to the subfamily Macropodusinae and is closely related to the genera *Betta* (Fighting fish) and *Macropodus* (paradise fish) (reviewed by Nelson et al. 2016*). Betta* and *Macropodus* lack a specialized sonic organ but possess a more sophisticated visual signalling system than *Trichopsis*. Representatives of *Betta* and *Macropodus* spread their opercula and gill membranes as well erect their unpaired fins during agonistic interactions. Together with a more colourful body colouration, they exhibit a repertoire of visual displays unknown in *Trichopsis* (Bischof 1996). *Macropodus* beats the pectoral fins similarly to croaking gouramis but does not produce sounds. Bischof (1996), however, in a detailed investigation of agonistic behaviour in the paradise fish *M. opercularis*, infrequently observed the production of pulsed pectoral sounds at low sound levels. This indicates that the ancestor of the genus *Trichopsis* started to emit sounds with unspecialized pectoral fin tendons and muscles and subsequently with enhanced tendons and muscles as soon as these incidental sounds became behaviourally significant. In contrast, *Trichopsis* did not evolve as many visual signals as the closely-related genera. This development of sonic structures may have started in all males, whereas females did not enhance tendons and muscles in all *Trichopsis* species.

A process in which an existing structure is modified and takes over a second function is termed exaptation by Parmentier et al. (2017). Those authors argue that sonic mechanisms in fishes (and perhaps other vertebrates) are often the result of exaptation. Pectoral fin tendons and muscles in gouramis are initially devoted to swimming, hovering and rapid fin beating as an agonistic display and subsequently became a sound-generating organ in *Trichopsis.*

### Conclusion

The present study reveals that closely related fish species within the genus *Trichopsis* differ in the extent to which they signal acoustically and visually and that additional differences exist between males and females in signalling during agonistic interactions. Genera closely related to *Trichopsis* did not evolve a sonic organ (they may, however, produce sounds with unspecialized teeth), but evolved a large repertoire of visual displays. It remains to be investigated which ecological factors (eg light and noise conditions, predation) triggered the evolution of sonic organs in particular fish taxa but not in others. Which factors resulted in the evolution of differently developed sonic organs in females also remain open. This calls for detailed analyses of signalling behaviour in closely related species of more taxa to find answers to the question why sound communication did or did not evolve in fishes.

## Supplymentary material

Supplemental Material: Trichopsis schalleri 2 croaking sounds
